# Alcohol Abuse Drug Disulfiram Is Effective against Cyst Stages of Entamoeba histolytica Parasite

**DOI:** 10.1128/aac.00832-22

**Published:** 2022-10-18

**Authors:** Ishrya Sharma, Laura Farr, Shannon Moonah

**Affiliations:** a Division of Infectious Diseases & International Health, Department of Medicine, University of Virginia, Charlottesville, Virginia, USA

**Keywords:** *Entamoeba histolytica*, disulfiram, drug development, drug repurposing, protozoan parasites

## Abstract

New anti-Entamoeba histolytica multistage drugs are needed because only one drug class, nitroimidazoles, is available for treating invasive disease, and it does not effectively eradicate the infective cyst stage. Zinc ditiocarb (ZnDTC), a main metabolite of the FDA-approved drug disulfiram, was recently shown to be highly effective against the invasive trophozoite stage. In this brief report, we show that ZnDTC is active against cysts, with similar potency to first-line cysticidal drug paromomycin.

## INTRODUCTION

Parasitic infections contribute significantly to worldwide morbidity and mortality. New parasite drugs are urgently needed, especially those that can target more than one life cycle stage (1–4). Entamoeba histolytica is a pathogenic protozoan parasite that is a major cause of diarrheal disease globally (5). The life cycle of E. histolytica consists of 2 stages, existing as either an infectious (transmissible) cyst or invasive (disease-causing) trophozoite (6–9). Given the little progress in new drug development over the past 60 years, only a single drug class (the nitroimidazoles, such as metronidazole) is available to effectively treat invasive amebiasis (10–12). In addition, nitroimidazoles are not effective in eradicating luminal cysts, which persist in the gut lumen after treatment. Therefore, the treatment regimen is complicated, as an additional luminal cysticidal agent, such as paromomycin, is required to complete therapy to prevent spread and reinfection (5, 13). The lack of a single drug that can effectively treat both life cycle stages creates issues with compliance, potential relapse, and disease transmission.

Drug discovery and development is time-consuming, risky, and costly, especially for nonprofitable infectious diseases. Drug repurposing involves identifying new uses for already approved drugs (1). Repurposing drugs might help meet the need for new antimicrobial therapies, as the process offers a less expensive and faster way to develop new treatments. Disulfiram is an inexpensive FDA-approved drug to treat alcoholism, with a well-established safety and tolerance profile. Disulfiram is a prodrug that is converted to active metabolites. In the body, disulfiram is rapidly metabolized to diethyldithiocarbamate (ditiocarb [DTC]). DTC then chelates with zinc to form zinc-ditiocarb (zinc diethyldithiocarbamate [ZnDTC]). ZnDTC is widely distributed throughout the body, including the site of infection in the gastrointestinal tract (14, 15). Protein turnover is essential for cell viability. Here, we evaluated ZnDTC for cysticidal effects given that it (i) disrupts protein homeostasis like paromomycin, (ii) is highly effective against E. histolytica trophozoites, and (iii) can penetrate the pathogen cell wall (16–18).

**FIG 1:**
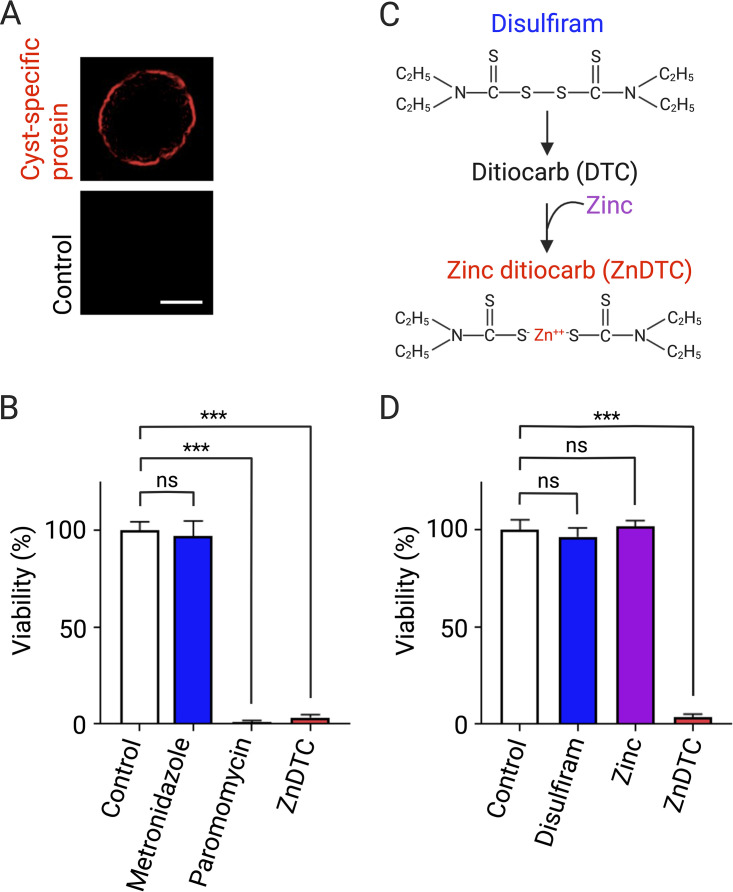
ZnDTC is active against E. histolytica cysts. (A) Immunofluorescence analysis with anti-E. histolytica cyst wall-specific Jacob protein or control antibodies. Scale bar, 5 μm. (B) Cysts were exposed to drugs at 10 μM for 72 h. (C) Illustration of how zinc ditiocarb (ZnDTC) is formed from disulfiram. (D) Evaluation of cysticidal activity of the prodrug disulfiram and active metabolite ZnDTC. Data represent mean and standard deviation of triplicates from 1 experiment and are representative of 3 independent experiments. ***, *P < *0.001; ns, nonsignificant.

**FIG 2:**
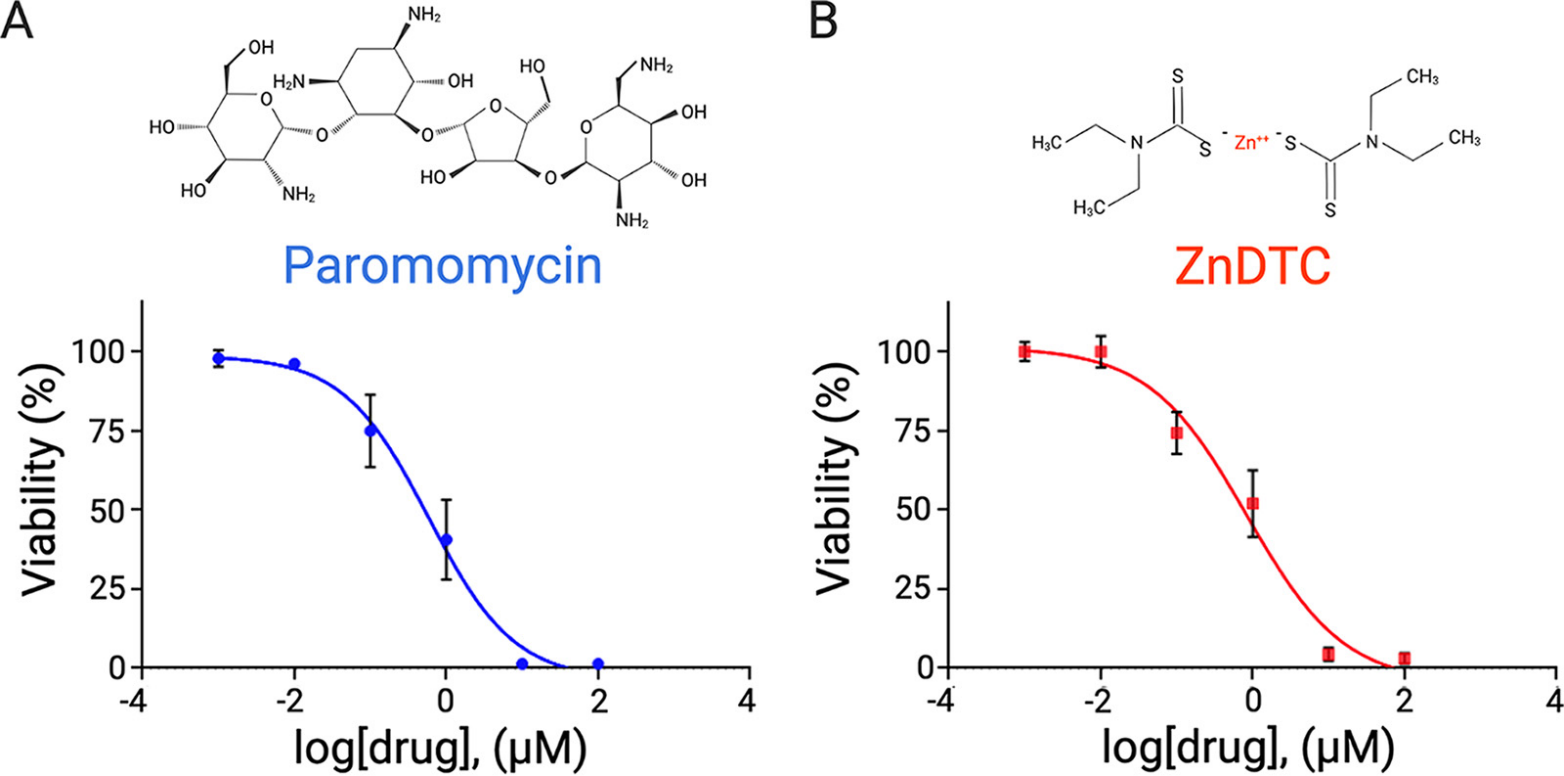
Comparison between paromomycin and ZnDTC cysticidal activities. (A and B) Dose-response curves showing cysticidal effect of paromomycin (A) and ZnDTC (B). EC_50_s for paromomycin and ZnDTC were 0.59 μM and 0.84 μM, respectively.

E. histolytica strains capable of surviving in the gut were used by passing trophozoites through mouse intestine. Cysts were generated as recently described (19). That is, trophozoites at high cell density of 50,000 cells/mL were cultured in glucose-free TYI (except for glucose from serum) containing the short-chain fatty acid butyrate. Detergent-resistant cysts were purified after treatment with 0.1% sarkosyl to remove any remaining trophozoites. Cysticidal activity was evaluated as previously described (20, 21), and cysts (20,000 per well) were treated with the pharmacological agents metronidazole, paromomycin, and ZnDTC at a concentration of 10 μM and vehicle control, including 0.1% dimethyl sulfoxide (DMSO), for 72 h at 37°C. Paromomycin, the drug of choice to treat ameba cyst, was used as a positive control. After drug exposure, cysticidal activity was measured using a dual fluorescent viability assay adapted from reference 22, which simultaneously detects fluorescein diacetate (FDA) and Sytox orange. Final concentrations of 5 μg/mL FDA and 0.5 μM Sytox orange were added together and read at 485/530 nm and 532/580 nm, respectively, after incubation at 37°C for 10 min. Statistical differences were determined using analysis of variance (ANOVA) followed by Dunnett’s *post hoc* test. A *P* value of less than 0.05 was considered statistically significant. Similar to paromomycin, we found that the disulfiram metabolite, ZnDTC, was significantly active against cysts. Consistent with previous findings, metronidazole had no effect on cyst viability. Additionally, unlike the active metabolite ZnDTC, the prodrug disulfiram did not demonstrate direct cysticidal activity (Fig. 1). Next, we determined the dose-response curve for the effect of ZnDTC on cyst viability. The compound was effective against parasites at submicromolar concentrations with similar potency to paromomycin, with 50% effective concentration (EC_50_) of 0.59 μM and 0.84 μM for paromomycin and ZnDTC, respectively (Fig. 2). Next, we determined the minimal cysticidal concentration (MCC). The MCC was defined as the lowest concentration of drug that resulted in no excystment and presence of trophozoite within 7 days of incubation (23). The MCCs for paromomycin and ZnDTC were at low micromolar concentrations, 3 μM and 7.5 μM, respectively. Entamoeba histolytica cysts are located in the lumen of the large intestine, which is lined by epithelial cells. We examined the cytotoxicity of ZnDTC on human intestinal epithelial cells (Caco-2) using CytoTox-One (Promega). ZnDTC demonstrated low toxicity (50% cytotoxic concentration [CC_50_] > 100 μM), consistent with its established safety profile.

Parasitic diseases like E. histolytica infection are in dire need of new drugs. Disulfiram is a drug that is already FDA approved, with a long history of safe clinical use. This study expands the antiparasitic potential of disulfiram and furthers the efforts to address this unmet medical need.
